# Noninvasive prenatal testing for the detection of fetal chromosome 17 microduplication: clinical implications and findings

**DOI:** 10.1186/s13039-024-00674-4

**Published:** 2024-04-22

**Authors:** Ye Shi, Fang-xiu Zheng, Jing Wang, Qin Zhou, Ying-ping Chen, Bin Zhang

**Affiliations:** Changzhou Maternal and Child Health Care Hospital, Changzhou Medical Center, Nanjing Medical University, China No. 16 Ding Xiang Road, Changzhou, 213003 Jiangsu Province China

**Keywords:** NIPT, 17q12 duplication, Karyotype, CMA, Prenatal diagnosis

## Abstract

**Background:**

Noninvasive prenatal testing (NIPT) is widely used to screen for fetal aneuploidies. However, there are few reports of using NIPT for screening chromosomal microduplications and microdeletions. This study aimed to investigate the application efficiency of NIPT for detecting chromosomal microduplications.

**Methods:**

Four cases of copy number gains on the long arm of chromosome 17 (17q12) were detected using NIPT and further confirmed using copy number variation (CNV) analysis based on chromosome microarray analysis (CMA).

**Results:**

The prenatal diagnosis CMA results of the three cases showed that the microduplications in 17q12 (ranging from 1.5 to 1.9 Mb) were consistent with the NIPT results. The karyotypic analysis excluded other possible unbalanced rearrangements. The positive predictive value of NIPT for detecting chromosomal 17q12 microduplication was 75.0%.

**Conclusions:**

NIPT has a good screening effect on 17q12 syndrome through prenatal diagnosis, therefore it could be considered for screening fetal CNV during the second trimester. With the clinical application of NIPT, invasive prenatal diagnoses could be effectively reduced while also improving the detection rate of fetal CNV.

## Background

Fetal chromosomal abnormalities are among the most important causes of neonatal birth abnormalities. Chromosomal microduplications or microdeletions are responsible for over 300 syndromes, which often show special facial appearance, mental development abnormalities, mental behavior abnormalities, growth retardation, autism, and so on [1]. Because of its high sensitivity and specificity, noninvasive prenatal testing (NIPT) is widely used in the prenatal screening of common fetal aneuploidies, including trisomies for chromosomes 13, 18, and 21 [2, 3]. NIPT can detect aneuploidies for all chromosomes because it involves the low-coverage whole-genome sequencing of maternal plasma cell-free DNA. Recent studies have reported that NIPT also can detect chromosomal microduplications or microdeletions [4].

This study aimed to investigate whether NIPT can be used to detect both copy number gains or losses. NIPT revealed copy number gains on chromosome in chromosome 17 in four fetuses. Further, karyotype analysis and chromosomal microarray analysis (CMA) were used to confirm the clinical efficiency of NIPT in detecting chromosomal copy number gains.

## Materials and methods

### Study participants

The study was conducted in accordance with the ethical guidelines of the Ethics Committee of the Changzhou Maternal and Child Health Care Hospital in Jiangsu province. The study participants consisted of four patients, with an average age of 33.25 ± 1.26 years and an average gestation age of 19.64 ± 1.60 weeks. Four cases showed copy number gains on the long arm of chromosome 17 at 17q12 by NIPT. Fetal developmental milestones and ultrasound findings showed no alterations throughout the pregnancies.

### Clinical procedure

All these pregnant women were offered genetic counselling and provided consent for prenatal diagnosis. The confirmatory (invasive) prenatal diagnosis via amniocentesis followed by karyotyping and CMA.

### Noninvasive prenatal testing

Whole blood sample (8 mL) of the study participants was collected in EDTA anticoagulant tubes. The samples were centrifuged within 8 h to extract the plasma. The plasma DNA was extracted from 1 ml plasma using a QIAamp Circulating Nucleic Acid Kit (Qiagen, USA). Then, low‐pass (× 0.1 genome coverage) massively parallel sequencing was performed on a NextSeq CN500 platform (Illumina, USA). The detailed technical procedure has been reported previously [5].

### Cytomolecular genetic analyses

Amniotic fluid (20 mL) was collected for chromosomal karyotype analysis using the G-banding technique. At least five karyotypes were analyzed and 20 metaphase cells were counted. DNA extracted from the amniotic fluid (10 mL) underwent CMA using the CytoScan 750 K Array (Affymetrix Inc., Santa Clara, CA, USA). The details have been reported previously [5].

### Follow-up

Information on the follow-up prenatal process and pregnancy outcomes was retrieved from the Jiangsu Provincial medical record system.

## Results

### Noninvasive prenatal testing

**Fig. 1:**
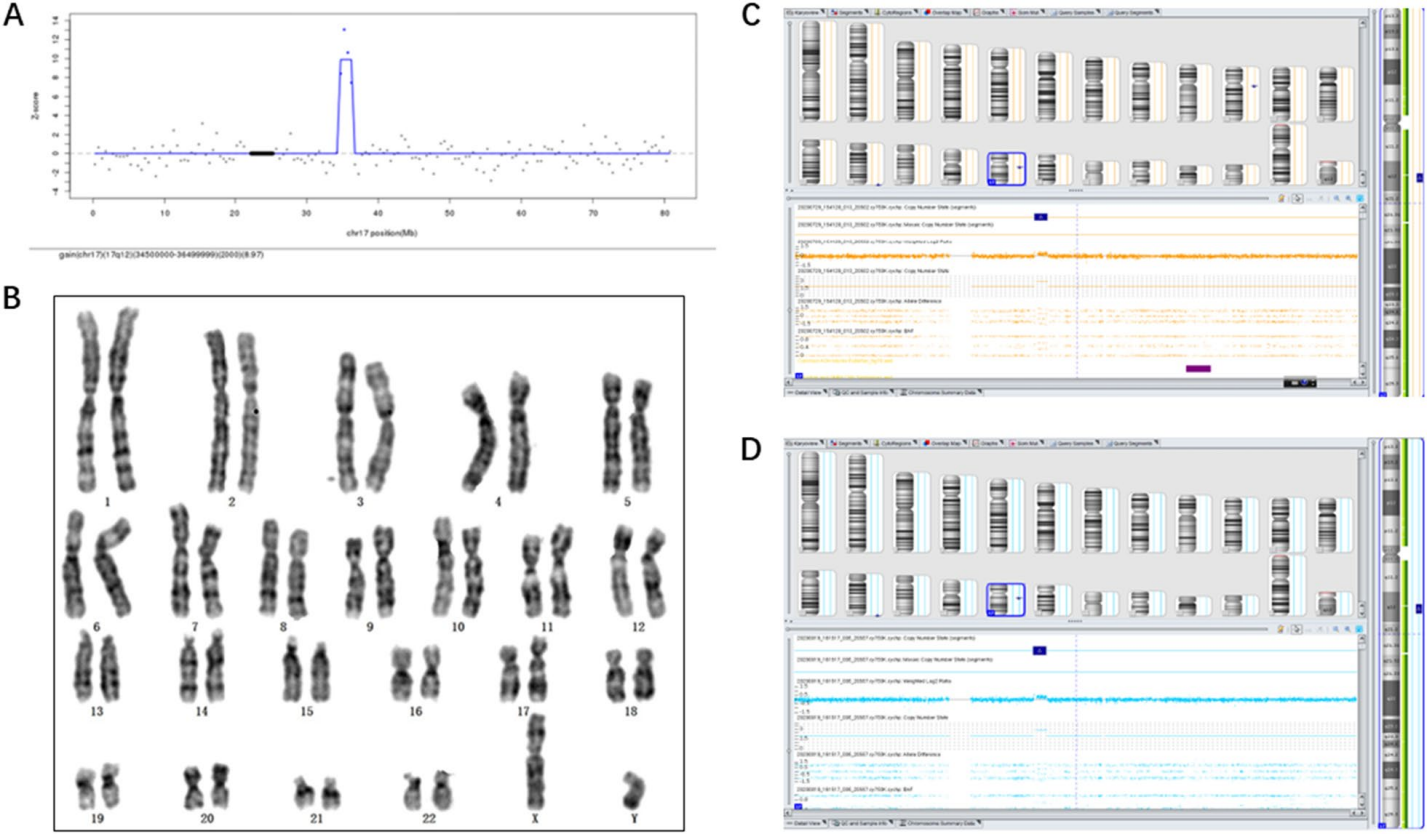
Overview of Case 1 from by NIPT, Karyotype analysis and CMA. **A** A NIPT study of maternal plasma showing a Z-score of 8.97 for fetal chromosome 17 and a duplication of 2 Mb from 34.5 to 36.5 Mb region. **B** Karyotype analysis of maternal amniotic fluid showing no significant fetal chromosomal abnormalities. **C** CMA analysis of amniotic fluid showing that a duplication of 1.9 Mb on chromosome 17q12 (arr [GRCh37] 17q12 (34,440,088–36,311,009) × 3). **D** CMA analysis of maternal peripheral blood showing that a duplication of 1.9 Mb on chromosome 17q12 (arr [GRCh37] 17q12 (34,405,514–36,307,773) × 3)

The Z-scores of chromosome 17 in the four cases ranged from 1.03 to 8.97. In addition, the NIPT results showed copy number gains of chromosome 17(2.0–3.0 Mb) (Table 1). Figure 1A visually demonstrates the NIPT result for Case 1, showing a Z-score of 8.97 for fetal chromosome 17, along with a 2 Mb duplication within the 34.5–36.5 Mb region.

**Table 1:** The results of NIPT, CMA and follow-up Outcomes

Case	Age	Gestational age	NIPT outcome	Prenatal Diagnosis (CMA)	Inheritance		Pregnancy outcome
					Maternal	Paternal	
1	32	19	gain(17q12) (34,500,000–36499999) (2.0 Mb)	arr[GRCh37] 17q12(34440088_36311009) × 3	arr[GRCh37] 17q12(34405514_36307773) × 3	46, XY	TOP
2	33	22	gain(17q12) (34,500,000–36499999) (2.0 Mb)	arr[GRCh37] 17q12(34822465_36351919) × 3	–	–	TOP
3	35	18 + 3	gain(17q12) (32,500,000–35499999) (3.0 Mb)	46, XX	–	–	Born, 40 + 1 weeks, female, 3740 g
4	33	19 + 1	X + Y, gain(17q12) (34,400,000–36499999) (3.0 Mb)	arr[GRCh37] 17q12(34421210_36351919) × 3	47, XXX	arr[GRCh37] 17q12(34421210_36351919) × 3	Born, 37 + 2 weeks, male, 3450 g

*TOP* Termination of pregnancy

### Karyotype analysis

Chromosomal analysis on amniotic fluid did not reveal any apparent structural chromosomal abnormalities in the fetuses.

### Chromosomal microarray analysis

The CMA results of the four cases were shown in Table 1. The prenatal diagnosis results of Case 1 (Fig. 1C), Case 2 and Case 4 were confirmed to differ slightly in terms of the size of the duplicated fragments and were consistent with those of the NIPT, but the result of Case 3 was a false positive. The positive predictive value (PPV) of NIPT, which detected a microduplication on chromosome 17q12, was 75.0%. Case 1 showed a duplication of approximately 1.9 Mb on chromosome 17q12, which encompasses 23 OMIM genes. Case 2 revealed a duplication of around 1.5 Mb on chromosome 17q12, containing 17 OMIM genes. Case 4 demonstrated a duplication of approximately 1.9 Mb on chromosome 17q12, harboring 27 OMIM genes.

### Pregnancy outcomes

After genetic counseling, both parents of case 1 and case 4 underwent CMA to investigate the mode of inheritance of this CNV and possibly its origin. However, the parents of Case 2 and Case 3 refused to undergo CMA, but based on the neonate's birth condition, Case 3 is considered a false positive. CMA results indicated that Case 1 inherited the alteration from its mother, whereas Case 4 inherited the alteration solely from its father. The parents of Case 1 and Case 2 chose to undergo termination of pregnancy. The other two cases chose to continue the pregnancy, and there have been no abnormalities in the growth and development of the children during follow-up after birth (Table 1).

## Discussion

Copy number variations (CNVs) in chromosome 17q12 include deletions and duplications. They are two different chromosomal aberrations, which are associated with a range of phenotypes. Deletions have been well-described, however, duplications are emerging as a novel genetic syndrome. The estimated prevalence of patients with 17q12 deletion was 1.6 per 1,000,000 citizens and that of patients with 17q12 duplication was 4.6 per 1,000,000 population in Denmark at the end of 2014 [6]. However, no other national prevalence data has been reported to date. Recurrent genomic rearrangements of the chromosomal region 17q12, ranging from ~ 300 Kb to ~ 2.1 Mb, which are pathogenic or likely pathogenic have been reported to be associated with various clinical phenotypes. Neurological symptoms are the most common features associated with 17q12 duplications, including learning disabilities, seizures, and structural brain anomalies [7–14]. The incomplete penetrance rate of this duplication is 21%. Typically, these conditions exhibit no significant phenotype during pregnancy or in early infancy. However, the use of NIPT can provide early risk warnings for 17q12 syndrome, allowing for earlier detection and intervention.

Current studies on 17q12 duplications are limited to phenotypic patients with 17q12 duplications or the family selected through the identification of 17q12 duplication in the proband. There have been no reports of prenatal screenings or diagnoses of 17q12 duplications. This may occur because detecting 17q12 duplications in fetuses is difficult. The current detection techniques for fetal chromosome microdeletions and microduplications are mainly FISH and CMA, which rely on invasive amniocentesis. As described above, neurological symptoms (including learning disabilities and delayed language development) are the most common features associated with 17q12 duplications (OMIM: #614526), which means that the ultrasound results usually show no significant abnormalities throughout the pregnancy, and invasive prenatal diagnosis is not recommended due to the absence of ultrasound indications. Although amniocentesis invasiveness has a less than 1% risk of abortion, there exist numerous contraindications to amniocentesis, such as threatened abortion, fever, bleeding tendency, uterine infection, and so on [15]. Women in these conditions cannot undergo amniocentesis. Therefore, more effective screening methods are required to detect the 17q12 duplication syndrome.

As a new prenatal screening method, NIPT has many advantages, including a simple and noninvasive operation and relatively easy quality control [16]. NIPT has been increasingly accepted by both clinicians and patients. It is efficient and accurate for the identification of the common fetal aneuploidies, especially for chromosomes 13, 18, and 21. Recently, some studies have found that NIPT, through deeper sequencing, can screen for microdeletions and microduplications, which are greater than 300 Kb in fetal genomes [17–21]. However, there have been no previous reports of screening the 17q12 duplication syndrome using NIPT.

In our study, we successfully used NIPT to detect copy number gains of approximately 2.0–3.0 Mb on fetal chromosome 17 in four patients. CMA was used to further delineate the specific duplication regions, with three of them confirming the results of NIPT. This chromosome 17q12 duplication syndrome would have been missed if these patients had not chosen NIPT for prenatal testing, which means that NIPT may be used to identify chromosome abnormalities in the fetuses. This study indicates that NIPT is efficient at detecting chromosomal microduplications. In addition, it should also be noted that one of the four cases was a false positive. Previous studies have identified a variety of potential causes of false‐positive NIPT, including confined placental mosaicism (CPM), maternal mosaicism, vanished twin, maternal tumors, and maternal copy number variation (CNV) [5].

Figure 1B displays the karyotype result for Case 1, indicating that chromosome 17 appears structurally normal on gross inspection. However, karyotype analysis has limitations, particularly in detecting smaller chromosomal changes or subtle rearrangements. This is where the NIPST result becomes crucial. As shown in Fig. 1A, the NIPS result for Case 1 revealed a significant finding: a 2 Mb duplication within the 34.5–36.5 Mb region of fetal chromosome 17. This duplication, although not visible on the karyotype, is a potential abnormality that may have implications for the fetus's health. Figure 1C further illustrates the abnormality in the CNV region, confirming the NIPS findings. Given the limitations of karyotype analysis, the NIPS result should be carefully considered for further diagnostic testing, such as CMA, to fully understand the implications and potential risks for the fetus.

Chromosomal region 17q12 gains, either pathogenic or likely to be so, have been associated with a range of clinical phenotypes. A crucial aspect of these gains is their incomplete penetrance, meaning that not all carriers will develop the associated condition. This incomplete penetrance significantly complicates clinical decision-making, particularly when considering pregnancy termination. Genetic counseling is essential in this regard, as it equips families with the knowledge necessary to make informed decisions. It helps them understand the complexities of penetrance and the potential phenotypic outcomes. During genetic counseling, families are guided in balancing the best interests of the fetus, the mother's welfare, and their own preferences and values. Clinicians must meticulously consider these factors when making decisions. Additionally, urgent research is needed to gain a deeper understanding of the clinical significance of copy number changes in regions like 17q12 and other regions exhibiting incomplete penetrance. This knowledge will enhance our understanding and treatment of these complex genetic conditions.

Furthermore, some limitations of this study should also be noted. Only four cases showed copy number gains of 17q12 through NIPT, and the relatively small sample size may have limited the statistical power and generalizability of the findings. Although two babies were born, the limited duration of this study may have constrained the ability to observe the clinical phenotypes of 17q12 syndrome, preventing discussion about incomplete penetrance.

## Conclusions

After prenatal diagnosis, NIPT has demonstrated effective screening for the 17q12 syndrome. The use of NIPT for detecting fetal copy number variations (CNVs) by analyzing cell-free fetal DNA (cffDNA) in the plasma of pregnant women will be rapidly adopted in clinical practice. Through the clinical application of NIPT, it can effectively reduce invasive prenatal diagnoses and enhance the detection rate of fetal chromosomal abnormalities, including CNVs.

## Data Availability

The datasets used and/or analyzed in the present study are available from the corresponding author upon reasonable request.

## Abbreviations

NIPT: Noninvasive prenatal testing
CMA: Chromosomal microarray analysis
CNV: Copy number variation
